# Inhibiting DNA Methylation Improves Survival in Severe Sepsis by Regulating NF-κB Pathway

**DOI:** 10.3389/fimmu.2020.01360

**Published:** 2020-07-02

**Authors:** Luxi Cao, Tingting Zhu, Xiabing Lang, Sha Jia, Yi Yang, Chaohong Zhu, Yucheng Wang, Shi Feng, Cuili Wang, Ping Zhang, Jianghua Chen, Hong Jiang

**Affiliations:** ^1^Kidney Disease Center, College of Medicine, The First Affiliated Hospital, Zhejiang University, Hangzhou, China; ^2^Key Laboratory of Nephropathy, Hangzhou, China; ^3^Kidney Disease Immunology Laboratory, The Third-Grade Laboratory, State Administration of Traditional Chinese Medicine of China, Beijing, China; ^4^Key Laboratory of Multiple Organ Transplantation, Ministry of Health of China, Hangzhou, China; ^5^Institute of Nephropathy, Zhejiang University, Hangzhou, China

**Keywords:** DNA methylation, sepsis, CLP, NF-κB pathway, DNMTs, inflammatory cytokine

## Abstract

Organ dysfunction caused by sepsis is life-threatening and results in high mortality. Therapeutic options for sepsis are limited. Pathogenic factors are considered as components of environmental pressure that modify DNA methylation patterns thereby enhancing disease progression. Here, we found that sepsis patients exhibited higher levels of genomic DNA methylation patterns and hypermethylated genes associated with the NF-kB signaling pathway. Therefore, we hypothesized that a DNA methyl transferase inhibitor, Decitabine, may mitigate inflammation and improve survival by inhibiting the NF-κB signaling pathway. To test the hypothesis, mice challenged with caecal ligation and puncture (CLP) were subcutaneously injected with Decitabine solution (0.5, 1, and 1.5 mg/kg) 2 h following operation. Our results indicated that Decitabine reduces DNA methyltransferases (DNMTs), attenuates NF-κB activation, downregulates inflammatory cytokine levels, and inhibits the progression of sepsis. Thus, DNA methylation may be indispensable for sepsis and serve as a predicting factor. The use of Decitabine could represent a novel strategy in the treatment of sepsis.

## Introduction

Sepsis, a life-threatening condition leading to organ dysfunction caused by dysregulated host response to infection, is a major cause of mortality among critical ICU patients (1). The annual incidence rate of sepsis ranges from 437 to 1,031 per 100,000, while its hospital mortality rate ranges from 25 to 30% in high-income countries (2–6). It significantly increases mortality rates among critical patients and also causes long-term sequelae, including cognitive and physical damage, in those who are discharged, thereby increasing their risk of death (7–9). Although sepsis is characterized by the systemic inflammatory response syndrome (SIRS) as well as the compensatory anti-inflammatory response syndrome (CARS) (10–13), its pathophysiology is complicated and its mechanism remains unclear. Aging is a high risk factor for sepsis outcome. A nationwide study, using a Cox proportional hazard model adjusting for comorbidity conditions, has shown that the incidence of sepsis in patients are older than 85 have an increased risk of mortality by ~31 times (14).

DNA methylation is an enzyme-induced, heritable process which regulates gene expression and affects the state of associated signaling pathways (15, 16). Under normal physiological conditions, methylation occurs in a highly specific manner during tissue-specific differentiation, and serves as a long-term memory of previous gene expression decisions to stabilize function (17). Under pathological conditions, gene activation or repression caused by abnormal demethylation or methylation, may lead to abnormal genetic expression and cause disease progression. DNA methylation is also a central element in immune system differentiation and function, allowing an appropriate gene expression pattern in immune cells (18–20). Pathogenic factors were considered as environmental pressure that could cause epigenetic changes (21). In addition, methylation index value increases in rapidly aging models and this can be used to determine the biological age of organisms (22), which may be related to the high incidence and mortality rate of sepsis patients. Several studies have revealed the involvement of epigenetic changes in sepsis, which may affect prognosis and serve as a diagnostic biomarker (23, 24). A recent study profiling 68 septic and 66 non-septic patients also revealed the presence of 668 differentially methylated regions (DMRs), that correlated with sepsis status (25). However, whether inhibition of DNA methylation has any influence on sepsis is yet to be determined.

Genome-wide methylation profiling conducted by the current study revealed that patients with sepsis displayed a DNA methylation pattern that was different from that displayed by the healthy. Utilizing RNA-sequencing results, we screened for decisive signaling pathways associated with sepsis. DNA methylation and RNA transcription axes may be the mechanisms underlying sepsis progression.

## Materials and Methods

### Patients and Blood Samples Collection

The studies involving human participants were reviewed and approved by the Research Ethics Committee of the First Affiliated Hospital, College of Medicine, Zhejiang University. The patients/participants (or their next of kin) provided written informed consent to participate in this study. Patients over 18 years, diagnosed with sepsis at the intensive care unit (ICU) were included in the study. These patients had been hospitalized at the First Affiliated Hospital, College of Medicine, Zhejiang University between 1 January 2015 and 20 May 2015. Sepsis was defined according to specific criteria of the American College of Chest Physicians/Society of Critical Care Medicine (26). Detail information of the selected patients was further reviewed by a clinician. Blood samples were obtained within the first 72 h following admission. Mononuclear cell separation was done within the first 24 h following collection. Controls were age matched healthy volunteers. DNA was extracted with QIAamp® DNA Mini and Blood Mini kits (Axygen, NY, USA). RNA was isolated via TRIzol® (Invitrogen, CA, USA).

### Infinium Human Methylation450 BeadChip

Five sepsis patients and two donors (control) were chosen for Genome-wide methylation profiling. DNA was extracted from peripheral mononuclear cells and quantitated. CpG sites with detected *p* < 0.01 and probe beads <3 in ≥5% samples, as well as 65 internal control SNP loci were filtered. Samples with filtered probes proportion >5% were abandoned in subsequent analyses. Methylation variable positions (MVP) were screened using the R limma package. Multiple hypothesis testing was conducted using the Benjamini & Hochberg method. Statistical significance was set at *p* < 0.05. R heatmap.2 package was used for heatmap drawing and the ggplot2 package was used for volcano plots.

### Illumina Hiseq PE150 Human Transcriptome Sequencing

Nine sepsis patients and three control donors were chosen for RNA sequencing. Of these, five sepsis patients died while four survived sepsis. After extracting total RNA from samples, DNA was digested using DNase I. The mRNA was enriched by magnetic beads carrying Oligo (dT) and broken into short fragments at a suitable temperature via a disruption reagent in a Thermomixer. Single-stranded cDNA was synthesized using interrupted mRNA as a template, which was then synthesized into double-stranded cDNA. Following purification and sticky end repair, adenine was added to the 3′ end of cDNA and ligated to the linker. Fragments were selected based on size and amplified via PCR. The established library was qualified using an Agilent 2100 Bioanalyzer and ABI StepOnePlus Real-Time PCR System. Subsequently sequencing was performed using an Illumina HiSeq^TM^ 2500.

Quality control was performed to clean data and determine whether the sequencing data was suitable for subsequent analysis. Clean reads were compared to the reference sequence with Bowtie2. After comparison, distribution and coverage of the reads on the reference sequence were analyzed to perform alignment QC. RPKM method (reads Per kb per Million reads) was used to estimate gene expression levels.

FPKM = 10^6^C/(NL/10^3^)

FPKM(A) is the expression level of gene A. C is the number of fragments that are uniquely aligned to gene A. N is the total number of fragments that are uniquely aligned to all genes, and L is the length of gene A (number of bases).

Bioconductor was used for differential gene expression analysis. The edge R function assumes that sequencing read counts for each gene follow the negative binomial distribution. Thus, hypothesis testing was based on this distribution. Screening conditions for significantly differentially expressed genes were FDR ≤ 0.05 AND |log_2_Ratio|≥1.

### Cecal Ligation and Puncture (CLP) Model and Survival Curve

All mice were maintained in the animal center of Zhejiang University according to animal care regulations. Research Ethics Committee of the First Affiliated Hospital, College of Medicine, Zhejiang University approved the experimental protocols. All experiments were carried out in accordance with the NIH Guide for the Care and Use of Laboratory Animals. Six- to eight-weeks old C57BL/6 wild type male mice were used for modeling. Polymicrobial sepsis was induced via CLP as previously described (27). Briefly, abdominal anesthesia was performed with 1% sodium pentobarbital (80 mg/kg). Each abdomen was disinfected using 75% alcohol, and the skin was paralleled on the right side of the midline of the abdomen (operator's field of vision). The cecum was exposed, ligated with 6/0 line and punctured using a 22 G needle from one side of the mesorectum to the contralateral side. One perforation of the caecum was performed. The cecum was returned to the abdominal cavity, and the abdominal muscle layer and skin were sutured layer by layer with a 4/0 line. The mice in the sham operation group were not subjected to cecal ligation and puncture, but all other procedures were the same as those for the experimental group. Two hours after CLP, the sham group and control group (CLP group) were subcutaneously injected with PBS. The sham+ DAC group was subcutaneously injected with 1 mg/kg Decitabine and mice in the CLP+ Decitabine groups were injected with different doses of decitabine solution (0.25, 0.5, 1, 1.5 mg/kg). Mice were housed in a clean animal room maintained at 22–26°C and observed for survival from 24 h to 7 d following surgery.

### Cytokine Antibody Array

In order to harvest spleen tissue, mice were sacrificed 6 h following CLP and spleens were stored at −80°C until further use. Mice spleens were homogenized using RIPA containing a protease and phosphatase inhibitor (Roche, Mannheim, Germany). Raybio® C-Series Custom Cytokine Antibody Arrays (Raybiotech, GA, USA) were performed according to the manufacturer's protocol. Briefly, a bicinchoninic acid (BCA) protein assay (Thermo, CA, USA) was used to measure and establish 500 μg of total protein for each sample. Membranes were incubated with blocking buffer for 30 min and aspirated. Diluted samples were then added to each assay well and incubated overnight at 4°C. Samples were aspired and the membranes were washed provided wash buffer I and II for a total of 5 times. Then biotinylated antibody cocktail was added into each well and incubated for 2 h at room temperature. The membranes were then washed 5 times. HRP-Streptavidin were pipetted into each well and incubated for 2 h at room temperature. After a third wash, detection buffer mixture was added onto each membrane following which the membranes were detected using chemiluminescence via Bio-rad ChemiDoc MP. The following 12 biomarkers were analyzed after positive control normalization and background subtraction; IL1b, IL1R1, TNF, TNFRSF1A, TNFSF14, TNFSF13b, Edar, Ticam1, CD40lg, CD40, CCL4, Icam1.

### RNA Extraction, cDNA Synthesis, and Real-Time Quantitative PCR

Total RNA was extracted from mice spleen tissues using TRIzol reagents (Invitrogen). PrimeScript™ II Reverse Transcriptase (Takara, Shiga, Japan) was used to reverse transcribe 1,000 ng of RNA to cDNA for each samples. Before qPCR, we conducted 1: 4 dilution of each samples. A two-step PCR reaction procedure was performed as follows: pre-denaturation, 95°C for 30 s; PCR reaction, 95°C for 5 s, annealing, 60°C for 31 s, 40 cycles; dissociation, 95°C for 15 s, 60°C for 1 min, 95°C for 15 s. The Ct value was taken as the average value of three replicate wells, and the Ct value of the β-actin PCR product was used as the internal reference, and the value obtained by comparing the Ct value of the PCR products of other genes was used as the relative expression value of the mRNA content. Specific primers for genes were designed via Primer-Blast (Supplementary Table 1). Real time-PCRs were carried out using SYBR Green and CFX96™ Real-Time PCR Detection Systems (Bio-rad, CA, USA).

### Western Blotting

Mice spleens were homogenized using RIPA containing a protease and phosphatase inhibitor (Roche). Nuclear proteins and cytoplasmic proteins were extracted from mice spleens using NE-PER™ Nuclear and Cytoplasmic Extraction Reagents (Thermo). Protein content was quantified using a Pierce™ BCA Protein Assay Kit (Thermo) and proteins were separated via SDS-PAGE with either 12.5%, 10% or 7.5% acrylamide and transferred into PVDF membranes (Millipore, Burlington, MA, USA). Next, blots were blocked with 5% milk or 5% BSA and probed with specific primary antibodies against phosphor-NF-κb p65, NF-κb p65, phosphor-IκBα, IκBα, phosphor-ERK, ERK (Cell Signaling Danvers, MA, USA), STK3/MST-2, phosphor- MST1/ MST2 (Abcam, Cambridge, UK), Phosphor-YAP, YAP, VEGF Receptor 2, Phosphor-VEGF Receptor 2, Phosphor-EGF Receptor, EGF Receptor (Cell Signaling, Danvers, MA, USA), DNMT1(Novus, Centennial, CO, USA), DNMT3a and DNMT3b (Abcam, Cambridge, UK) overnight at 4°C. The membranes were then washed with TBST and incubated with horseradish peroxidase-conjugated secondary antibodies at room temperature for 1 h. Next, target proteins were detected using chemiluminescence via Bio-rad ChemiDoc MP and normalized to β-actin or histone H3.

### Statistical Analysis

Statistical analyses were performed using GraphPad Prism 7.0 (GraphPad Software, CA, USA). Experiments were replicated at least 3 times with similar results. The data were expressed as mean ± SD. Student's *t*-tests were used for comparison between two groups. Survival data were presented as Kaplan-Meier plots and compared via Log-rank test. Significance was set at *P* < 0.05.

## Results

### Bioinformatics Analysis Suggested Up-Methylated NF-κB Signaling Pathway-Related Genes in Patients With Sepsis

**Figure 1:**
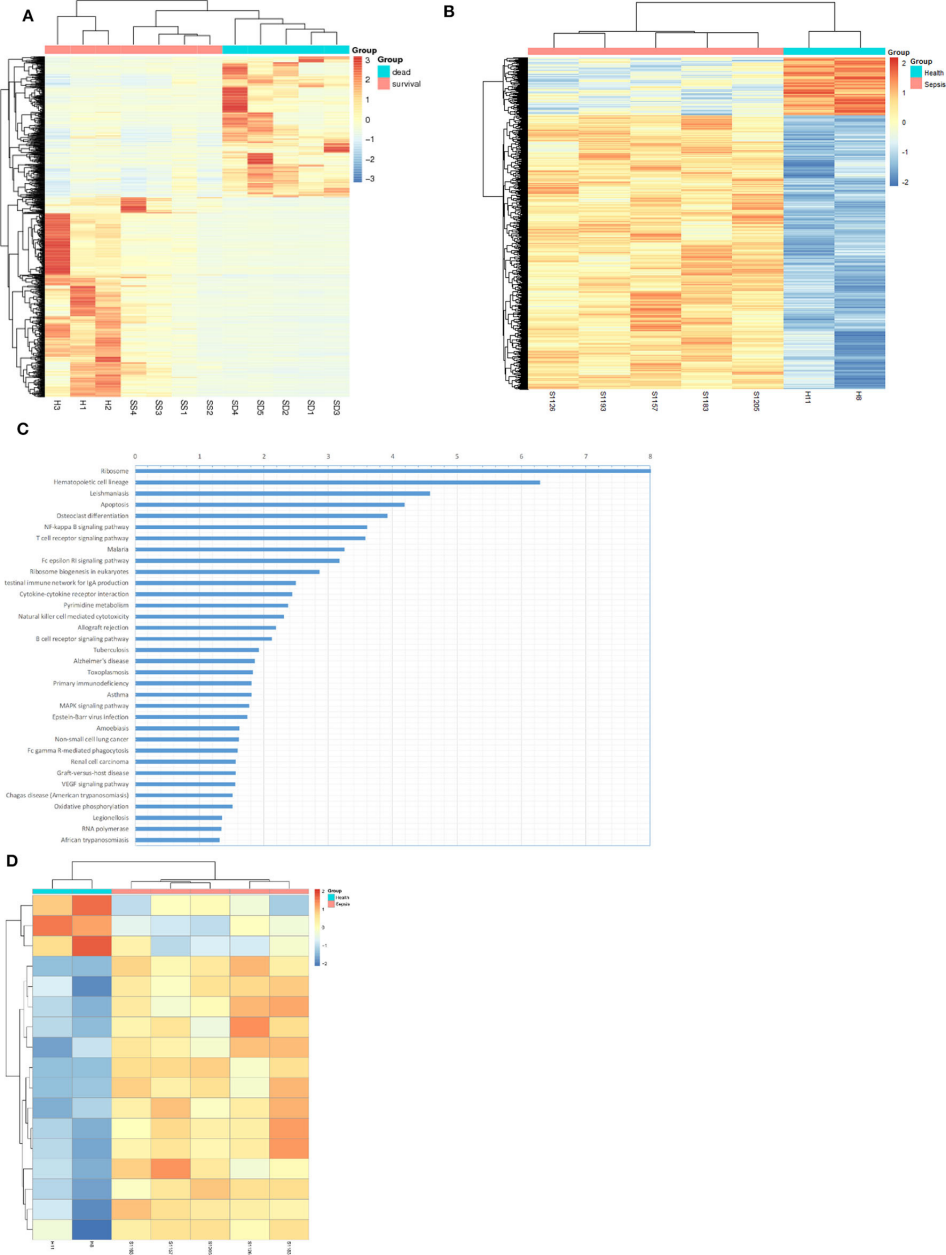
Bioinformatics analysis suggested up-methylated NF-KB pathway related genes in patients with sepsis. **(A)** Hierarchical clustering and heat map of differentially expressed genes in survival sepsis patients, dead sepsis patients, and control. The scaled expression value is shown in a blue-red color scheme with red indicating higher expression, and blue lower expression (FDR ≤ 0.05 AND |log_2_Ratio| ≥ 1). **(B)** Hierarchical clustering and heat map of methylation variable positions in sepsis patients and control. The scaled expression value is shown in a blue-red color scheme with red indicating up-methylated, and blue down-methylated (*p* < 0.01). **(C)** Kyoto Encyclopedia of Genes and Genomes (KEGG) enrichment analysis of DEGs (*p* < 0.05). **(D)** Hierarchical clustering and heat map of NF-κB pathway related genes in methylation variable positions (*p* < 0.01).

RNA sequencing and methylation profiling techniques combined with bioinformatics analysis were used to characterize differences between sepsis patients and healthy people. For the purpose of RNA sequencing analysis, we analyzed differential gene expression via unsupervised hierarchical clustering which is shown as an expression heatmap (Figure 1A). The hierarchical heatmap indicated tight clustering not only between sepsis patients and healthy individuals but also between sepsis patients who died and who survived. For methylation profiling, we determined methylation variable positions (MVPs) via unsupervised hierarchical clustering and produced a heatmap (Figure 1B). Of these MVPs, 4,066 (82.6%) were hypermethylated, while 859 (17.4%) were hypomethylated, indicating that sepsis patients exhibited a higher level of genomic DNA methylation pattern. In order to identify the altered pathways related to DNA methylation and gene transcripts, KEGG pathway analyses were carried out using the differentially expressed genes, wherein the NF-κB signaling pathway was the highest-ranked pathway (Figure 1C). When genes associated with the NF-κB signaling pathway were screened, we found that most of these genes (14/17) were hypermethylated in sepsis patients compared with that of the control (Figure 1D).

### Decitabine Improved Inflammatory Response and Survival in Mice With Severe Sepsis Induced by CLP

**Figure 2:**
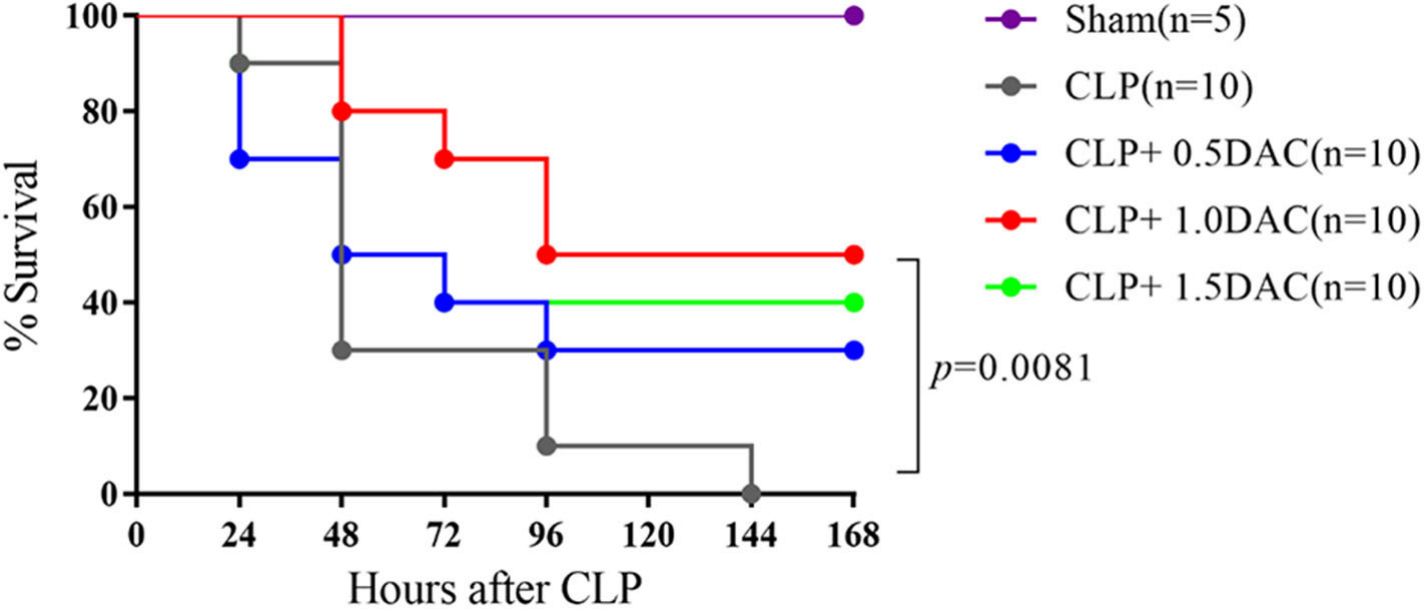
Decitabine improved survival in severe sepsis mice induced by CLP. Survival curve after CLP of mice treated with Decitabine. Mice were injected subcutaneously with either 0.5, 1.0, and 1.5 mg/kg Decitabine (CLP+ 0.5 DAC, CLP+ 1.0DAC, CLP+ 1.5DAC, *n* = 10) or PBS (CLP, *n* = 10) 2 h after CLP and were monitored for 7 days or until death. Sham (laparotomy without CLP) groups (*n* = 5) served as controls. CLP, cecal ligation and puncture; DAC, Decitabine; PBS, phosphate-buffered saline. *p* = 0.0081 by Log-rank test.

Considering the increased degree of methylation in sepsis patients, we investigated whether Decitabine, a DNMTs inhibitor, alleviates CLP-induced sepsis in mice. Pro-inflammatory cytokines, IL-6, IL-1β, and TNF-α, peaked at 6 h following CLP in our models (Supplementary Figure 1A). Acute lung damage is one of the main causes of death among sepsis patients. Severe lung injury was estimated using a score representing the presence of alveolar hemorrhage, edema, inflammation, and enhanced recruitment of neutrophils as well as leukocytes into the alveolar spaces at 6 h (11.8 ± 0.4472),12 h (9.2 ± 1.924), 18 h (7.6 ± 1.817), and 24 h (9.4 ± 1.517) following CLP (Supplementary Figures 1B,C). Therefore, 6 h after CLP was selected as the observation point. Mice were subjected to CLP-induced sepsis via either subcutaneous injection with different doses of Decitabine, or PBS, 2 h after CLP. Relative expression levels of IL-6, IL-1β, and TNF-α were lower in mice treated with 1 mg/kg and 1.5 mg/kg Decitabine (Supplementary Figure 2A). However, 1.5 mg/kg Decitabine could not further ameliorate inflammatory response. Next, animal survival was monitored up to 7 d post-operation or until death. No deaths occurred in the sham group. Under our sepsis model conditions, all mice died within 7 d after CLP induction. However, a dose of 1 mg/kg Decitabine significantly improved survival in mouse CLP-induced sepsis models (Figure 2). To determine whether Decitabine exerts a positive effect on lung injury among CLP-induced models, we further investigated lung morphology. Compared with the CLP group, 1.0 mg/kg (6 ± 0.7071) and 1.5 mg/kg (5.6 ± 1.14) Decitabine-treated mice exhibited less histological damage and lower scores (Supplementary Figures 2B,C). These results indicated that Decitabine protects against CLP-induced death by alleviating lung injury.

### Decitabine Degrades Intracellular DNMTs Protein Level in CLP Mice

**Figure 3:**
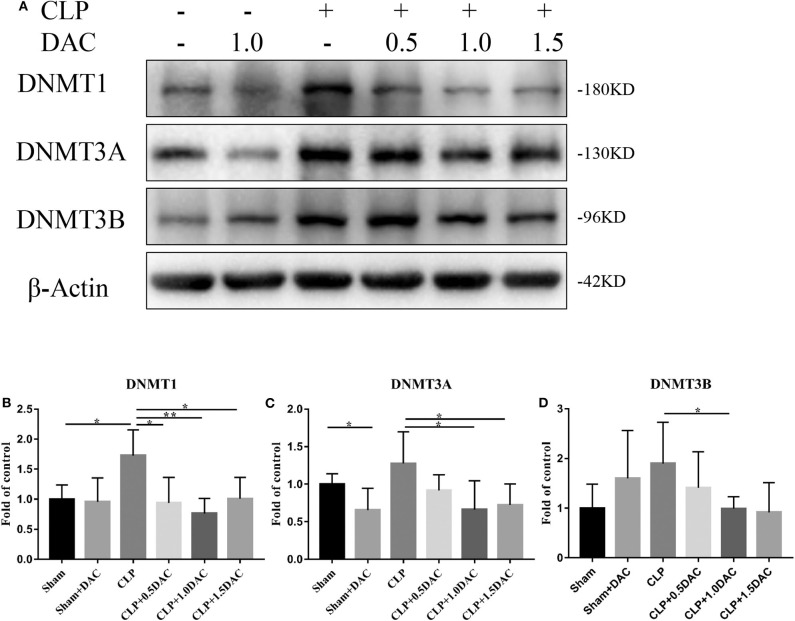
Decitabine degrade intracellular DNMT protein level in CLP mice. **(A)** Representative western blot for DNMT1, DNMT3A, and DNMT3B in mice spleen with or without Decitabine treatment. **(B–D)** The bands were quantified by densitometry and normalized to the density of β-actin *n* = 5. Data were shown in Mean ± SD. **p < 0.01, *p < 0.05. Comparisons between two groups were done by independent student's *t*-test.

Decitabine is known to degrade DNMT1, DNMT3A, and DNMT3B proteins in tumor tissue by inducing lysosomal degradation of DNMTs (28). To investigate the mechanism underlying sepsis models, spleen tissues were harvested and subjected to protein analysis. The levels of all 3 DNMTs in CLP-induced sepsis mice tended to increase compared to those of the sham group, but only DNMT1 showed a significant elevation (Figure 3). Interestingly, elevation of DNMT1, DNMT3A, and DNMT3B were all attenuated by Decitabine treatment upon different dose of Decitabine (Figure 3).

### NF-κB Activation in CLP Mice Was Attenuated Upon Decitabine Treatment

**Figure 4:**
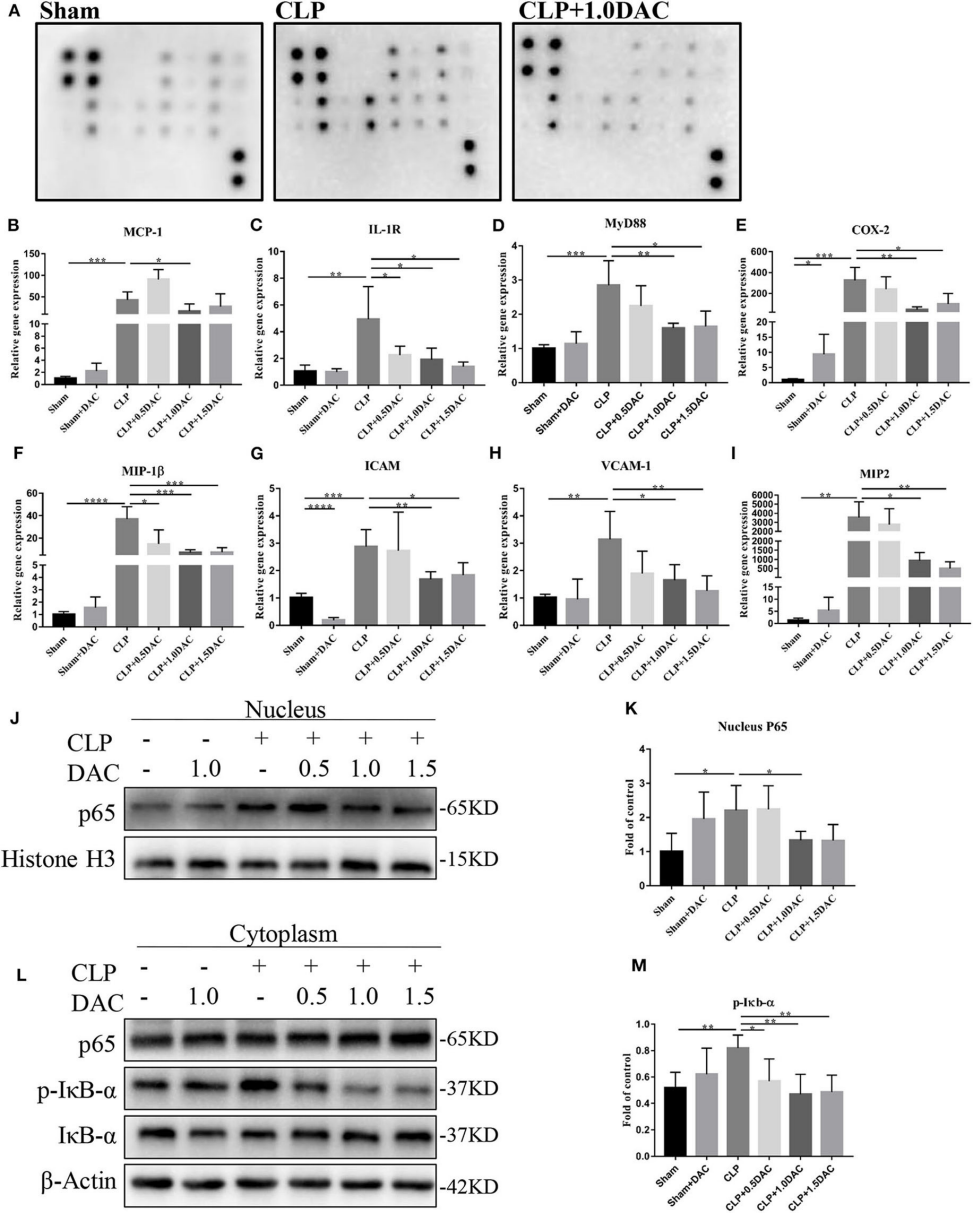
Decitabine attenuated NF-κB activation in CLP mice. **(A)** Representative images of cytokine expression levels in spleen lysate of sham, CLP and CLP+ 1.0DAC mice. **(B–D)** Relative mRNA levels of NF-kB upstream targets genes MCP-1, IL-1R, MyD88 in CLP mice spleen samples with or without Decitabine treatment compared with sham group spleen samples. **(E–I)** Relative mRNA levels of NF-κB downstream targets genes COX2, MIP-1β, ICAM, VCAM-1, MIP-2 in CLP mice spleen samples with or without Decitabine treatment compared with sham group spleen samples. **(J)** Representative western blot for P65 in nucleus fraction of mice spleen with or without Decitabine treatment. **(K)** The P65 bands were quantified by densitometry and normalized to the density of Histone H3. **(L)** Representative western blot for P65, p-IκB−α, IκB−α in cytoplasm fraction of mice spleen with or without Decitabine treatment. **(M)** The p-IκB−α bands were quantified by densitometry and normalized to the density of β-actin *n*=5. Data were shown in Mean ± SD. ****p < 0.0001, ***p < 0.001, **p < 0.01, *p < 0.05. Comparisons between two groups were done by independent student's *t*-test.

Bioinformatics analysis suggested that the NF-κB pathway was activated and up-methylated in sepsis patients (Figure 1). In order to further elucidate underlying mechanisms and verify whether Decitabine improved the symptoms and survival of CLP mice by inhibiting the NF-κB pathway, we applied the cytokine antibody array technique to identify the key factors associated with the NF-κB pathway. Twelve factors that satisfied the following were identified: [1] methylation variable positions in sepsis patients and control (*p* < 0.05); [2] differentially expressed genes in survival sepsis patients, dead sepsis patients and control (FDR ≤ 0.05 AND |log_2_Ratio|≥1); [3] core factors in the NF-κB pathway; and [4] included in the Raybio® C-Series Custom Cytokine Antibody Arrays list (Supplementary Figure 3A). Relative expression levels of these 12 cytokines were in accordance with bioinformatics analysis (Figure 4A; Supplementary Figure 3B). Next, we performed qPCR on mice spleens. NF-κB upstream targets genes, MCP-1, IL-1R, and MyD88, which were significantly upregulated in CLP-induced sepsis mice, were attenuated by Decitabine treatment (Figures 4B–D). It was observed that the NF-κB downstream targets genes, COX2, MIP-1β, ICAM, VCAM-1, and MIP-2, were also upregulated (Figures 4E–I). Interestingly, 1.5 mg/kg Decitabine could not further attenuate the expression of these target genes.

The first step of NF-κB activation involves post-translational modification of IκB. In the canonical pathway (CP), activation of the IκB kinase (IKK) complex leads to the phosphorylation of two specific serines near the N terminus of IκBα, which target IκBα for ubiquitination and degradation. This causes NF-κB dimers, which actively shuttle between the nucleus and cytosol, to stay in the nucleus and induce gene expression (29). Thus, nuclear proteins and cytoplasmic proteins were extracted separately to further validate the involvement of NF-κB in our models. Our data showed that nuclear P65, which was increased in CLP-induced sepsis mice, was significantly inhibited by Decitabine treatment (Figures 4J,K). cytoplasmic P65 levels did not differ between the 3 groups (Figure 4L). Consistent with nuclear P65, the increase in p-IκBbα during CLP-induced sepsis was significantly attenuated by the Decitabine treatment (Figures 4L,M). In accordance with the expression level of NF-κB upstream and downstream targets genes, 1.5 mg/kg Decitabine cannot further attenuate NF-κb pathway.

In order to demonstrate whether NF-κB was the core factor modified by altered methylation induced by sepsis, we verified 4 more signaling pathways, including the MAPK signaling pathway, Hippo signaling pathway, EGFR signaling pathway, and the ErbB signaling pathway, which were placed at the top in the KEGG of differentially expressed genes and methylation variable positions, (Supplementary Figure 4; Figure 1C). Our data showed that there were no significant differences in p-MST1/2, p-YAP, p-AKT, p-P38, p-EGFR, and VEGFR2 between the CLP group and the DAC group (Supplementary Figure 5). Considered together, these results indicated that the NF-κB signaling pathway was activated strongly 6 h following operation in CLP-induced sepsis mice, whereas Decitabine protected sepsis mice from such activation.

## Discussion

Sepsis is a life-threatening complication caused by bacterial infections, leading to a whole-body inflammatory response that causes unacceptably high mortality with limited therapeutic options (30, 31). Therefore, further improvement of treatment regimens intended for sepsis are needed. The current study explored the possibility of developing novel epigenetic therapies. We established a sepsis model by performing CLP on mice followed by Decitabine injection, in order to study the effect of DNA methylation inhibition. Decitabine is a DNA methyltransferase inhibitor, which has been found to be effective in some hematological diseases in addition to being well tolerated in older patients over 65 years of age (32, 33). Our data demonstrated that Decitabine notably improves survival, and that its beneficial effects may be attributed to the attenuation of DNMT levels, inflammatory cytokines expression, and NF-κB pathway activation.

DNA methylation chip sequencing results of sepsis patients and healthy controls indicated that high DNA methylation level sites were more concentrated in sepsis patients, while corresponding healthy controls showed a lower level of genomic DNA methylation patterning. A recent study profiling 68 sepsis and 66 non-sepsis patients also revealed 61% hypermethylated regions in 668 DMRs, 81% of the differentially methylated region–associated genes were differentially expressed in one or more datasets containing sepsis and non-sepsis patients (25). There is an extensive system of proteins in the cells involved in writing the methylation pattern on DNA via *de-novo* methylation (DNMT3A and DNMT3B) and copying methylation patterns during DNA replication via methylation maintenance (DNMT1) (17). Reportedly, circulating extracellular vesicle DNMTs are highly correlated with sepsis severity and may serve as a predicting factor (34). As observed during the current study, DNMT1 was upregulated in CLP mice, which substantiated the findings of a previous study on LPS endotoxic shock models (35). Thus, we speculated that the hyper-methylation pattern seen in sepsis may mainly be attributed to modified maintenance methylation. Decitabine first inserts into the DNA sequence by replacing cytosine, forming a covalent bond between the 5-aza-cytosine ring and DNMT1. DNA replication proceeds in the absence of DNMT1, resulting in the loss of DNA methylation in the nascent strand (36). Our studies revealed another mechanism of Decitabine, involving the reduction of DNMT levels in sepsis mice. Another study performed on multiple breast cancer cell lines and PDX tumor tissues suggested that this latter mechanism may involve proteosomal-dependent degradation (28).

RNA sequencing analysis not only revealed a tight clustering between sepsis patients and healthy individuals but also between survived and non-survived patients with sepsis, consistent with previous studies (37, 38).

KEGG pathway analysis of transcriptome results revealed the important role played by the NF-κB pathway in sepsis. DNA methylation levels of differential sites associated with the genes in NF-κB pathway was higher in sepsis patients than those in the healthy controls. Previous studies have demonstrated that DNA methylation can regulate NF-κB activation in different ways, such as by inhibiting binding of the kappa B site to transcription factors (39), or regulating miRNAs upstream of NF-κB (40). Thus, we intend to explore the role of DNA methylation in sepsis by studying the mechanism regulating the DNA methylation-NF-κB pathway.

The pathophysiological process of sepsis involves a complex network of cytokines and inflammatory mediators. Inflammatory responses may be activated by pathogenic microorganisms and the by-products of these organism via the transcription of inflammatory factors, resulting in the release of a large number of inflammatory mediators. Our pre-experiment indicated that the inflammatory factors, IL-6, IL-1β, and TNF-α, in CLP mice increased significantly and reached a peak at 6 h, followed by a decrease at 12, 24, and 30 h. We found that the relative gene expression of inflammatory factors, IL-1β, IL-6, and TNF-α and MCP-1, as well as the 12 factors detected via the antibody array, such as TNFR1 and CD40, was down-regulated in CLP mice following treatment with Decitabine. TNF-α and IL-1β are two important mediators of septic shock that affect endothelial function, such as regulation of vascular tone, vessel permeability, and leukocyte exudation (41). Multi-microbial sepsis induced by the CLP procedure may cause a significant increase in intestinal permeability, which is significantly improved in IL-6-deficient mice (42). TNFR1-deficient mice, compared with B6 mice, survived lethal polymicrobial infection with enhanced neutrophil recruitment and bacterial clearance in the peritoneal cavity (43). Anti-CD40 treatment provided nearly complete protection against sepsis-induced lymphocyte apoptosis, and improved survival in sepsis (44). Although these genes played an important role in host defense against invading bacteria, they caused SIRS and septic shock once overproduced. Therapeutic strategies that attenuate the inflammatory response may improve survival.

In the current study, transcriptional activation of NF-κB upstream and downstream genes MCP-1, IL-1R, MyD88, ICAM, VCAM-1, MIP-1β, MIP2, and COX2, was significantly downregulated in CLP mice following the administration of Decitabine. Similarly, Decitabine treatment downregulated the expression of inflammation genes, TNF-α, IL-6, and IL-1β, and the chemotaxis gene MCP-1 resulting in the amelioration of atherosclerosis (45). The presence of Decitabine in cultures promotes potent down-regulation of COX-2 and MIP-2 genes in tumor-derived myeloid cells (46). In addition, antibody array is a powerful tool that can be used to determine potential targets involved in sepsis. The 12 factors in the antibody array showed tendencies that were similar to those seen in the qPCR data. Furthermore, Decitabine markedly reduced phospho-IκBα and nuclear NF-κB-p65, strongly suggesting that Decitabine inhibits activation of the NF-κB pathway.

NF-κB activation may be caused by numerous pathogenic microorganisms and lead to the activation of these cytokine networks, thereby mediating the transcription of a large number of genes that promote sepsis progression (47). NF-κB activity was significantly higher in patients who died than that in patients who survived (48). Animal studies have demonstrated the important role played by NF-κB in the pathophysiology of sepsis. Blocking the NF-κB pathway could improve systemic hypotension, adjust myocardial and vascular function, inhibit the expression of numerous pro-inflammatory genes, improve intravascular coagulation, reduce neutrophil infiltration in tissues, and prevent microvascular endothelial leakage. In rodent sepsis models, inhibition of NF-κB activation could prevent multiple organ injury and improve survival rate. In animal models of sepsis established by injecting LPS, or CLP surgery, studies have demonstrated the protective effects of different NF-κB inhibitors on animals with sepsis (49–51). In CLP mice, inhibiting IKK activity reduced cytokine production and alleviated systemic hypotension (52). However, compounds acting directly on the NF-κB pathway, such as IKK inhibitors, also exerted a role independent of NF-κB, leading to a high risk of side effects and toxicity, despite having a significant inhibitory effect on inflammatory cytokines (53, 54). We administered Decitabine to CLP mice in order to investigate its effect on sepsis and found that the survival rate improved. This aroused our interest in investigating this phenomenon. Some studies have reported that accelerated degradation of p65 protein may provide a solution for inflammation (55).

**Figure 5:**
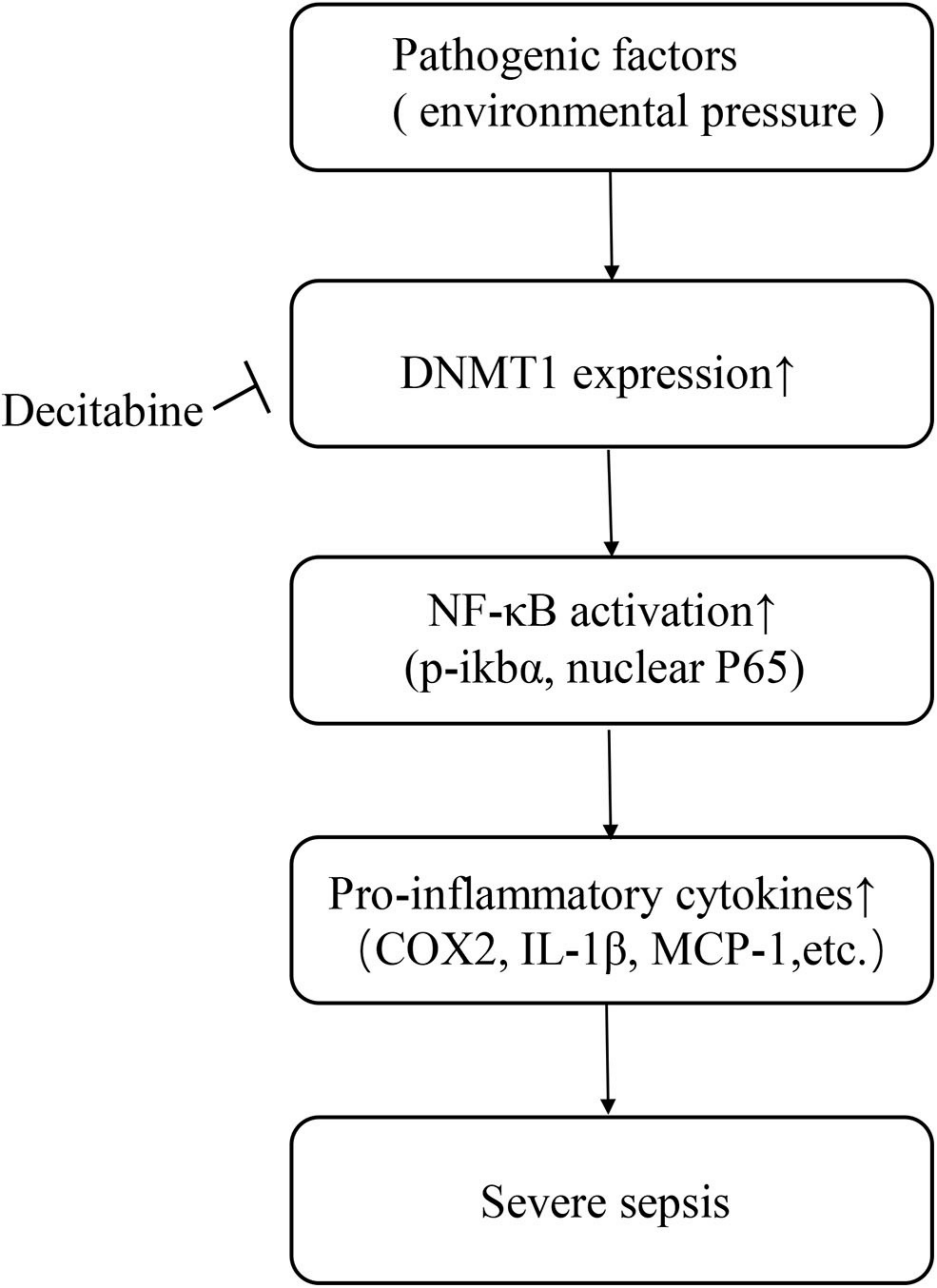
Schematic of study.

Overall, our data indicates that the DNA methylation pattern may serve as a possible predictive factor of sepsis. However, these findings need to be further defined via randomized studies involving larger numbers of patients. The current study also revealed a potential therapeutic targeting the DNA methylation-NF-κB pathway axis in sepsis. Briefly, pathogenic organisms are considered as an environmental pressure related factor that modifies DNA methylation patterns by upregulating intracellular DNMTs, especially DNMT1. Activation of the NF-κB pathway increases the expression and release of inflammatory cytokines, leading to tissue injury and disease progression (Figure 5). A single dose of Decitabine administered following CLP reduced DNMTs, attenuated NF-κB activation and thereby downregulated inflammatory cytokine levels and inhibited the progression of sepsis. However, further research on optimizing the timing and dosage of drug application intended to optimize the protective effect of Decitabine is needed. Considering the wide range of effects exerted by Decitabine, it is believed that development of inhibitors for certain methylation sites may be more beneficial. Further studies focused on specific DNA methylation sites of the NF-κB pathway that are involved in sepsis may also be of significance.

## Data Availability Statement

The raw data supporting the conclusions of this article will be made available by the authors, without undue reservation, to any qualified researcher.

## Ethics Statement

The studies involving human participants were reviewed and approved by Research Ethics Committee of the First Affiliated Hospital, College of Medicine, Zhejiang University. The patients/participants (or their next of kin) provided written informed consent to participate in this study. The animal study was reviewed and approved by Research Ethics Committee of the First Affiliated Hospital, College of Medicine, Zhejiang University.

## Author Contributions

LC, TZ, and XL carried out experiments. SJ and YY analyzed bioinformatics. CW carried out mice model. CZ, YW, and SF analyzed pathology. PZ contributed to clinical assessment. JC and HJ conceived and supervised the study. XL and TZ drafted the manuscript. All authors were involved in writing the paper and approved the final version.

## Conflict of Interest

The authors declare that the research was conducted in the absence of any commercial or financial relationships that could be construed as a potential conflict of interest.

## Supplementary Material

Supplementary Figure 1 **(A)** Quantitative PCR analysis of spleen IL-6, IL-1β, TNF-α at different time after CLP shows the expression of these factors reached the highest peak 6h after CLP. **(B)** The mice were sacrificed at different time after CLP and lung tissue was stained with haematoxylin-eosin (magnification, × 400). **(C)** The lung injury score. *n* = 5. Data were shown in Mean ± SD. ^****^*p* < 0.0001, ^***^*p* < 0.001, ^**^*p* < 0.01, ^*^*p* < 0.05. Comparisons between two groups were done by independent student's *t*-test.

Supplementary Figure 2 **(A)** Quantitative PCR analysis of spleen IL-6, IL-1β, TNF-α in different dose of Decitabine treated mice. **(B)** Effects of Decitabine on CLP lung morphology. The mice were sacrificed 6 h after operation and lung tissue was stained with haematoxylin-eosin (magnification, × 400). **(C)** The lung injury score. *n* = 5. Data were shown in Mean ± SD. ^****^*p* < 0.0001, ^***^*p* < 0.001, ^**^*p* < 0.01, ^*^*p* < 0.05. Comparisons between two groups were done by independent student's *t*-test.

Supplementary Figure 3 **(A)** Location of cytokine antibody spotted onto the membrane. **(B)** Relative expression of 12 cytokines in mice spleen lysate *n* = 3. Data were shown in Mean ± SD.

Supplementary Figure 4 Kyoto Encyclopedia of Genes and Genomes (KEGG) enrichment analysis of methylation variable positions (*p* < 0.05).

Supplementary Figure 5 **(A)** Representative western blot for p-JNK, JNK, p-MST1/2, MST2, p-AKT, AKT, p-P38, P38, p-EGFR, EGFR, VEGFR2, and β-Actin in sepsis mice spleen with or without Decitabine treatment. **(B–G)** The bands were quantified by densitometry and normalized to the density of corresponding total protein or β-actin *n* = 3.Data were shown in Mean ± SD. ^****^*p* < 0.0001. Comparisons between two groups were done by independent student's *t*-test.
