# Supplementary figures and images for: Inhibiting DNA Methylation Improves Survival in Severe Sepsis by Regulating NF-κB Pathway
Source: Front Immunol. 2020 Jul 2;11:1360. doi: 10.3389/fimmu.2020.01360 (PMC7343767; doi:10.3389/fimmu.2020.01360)

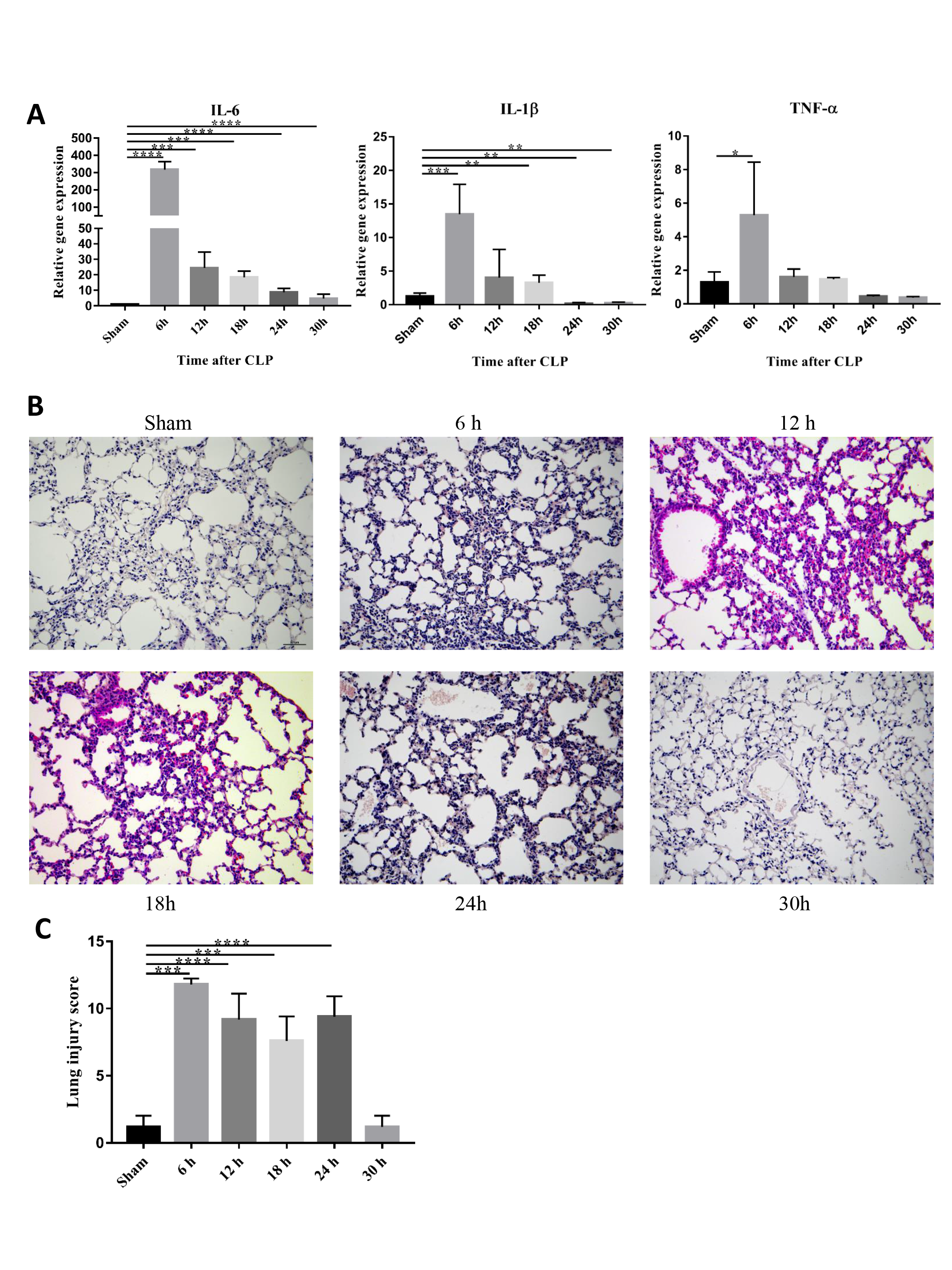

Supplement: Supplementary Figure 1 — (A) Quantitative PCR analysis of spleen IL-6, IL-1β, TNF-α at different time after CLP shows the expression of these factors reached the highest peak 6h after CLP. (B) The mice were sacrificed at different time after CLP and lung tissue was stained with haematoxylin-eosin (magnification, × 400). (C) The lung injury score. n = 5. Data were shown in Mean ± SD. ****p < 0.0001, ***p < 0.001, **p < 0.01, *p < 0.05. Comparisons between two groups were done by independent student's t-test. [file Image_1.TIF]

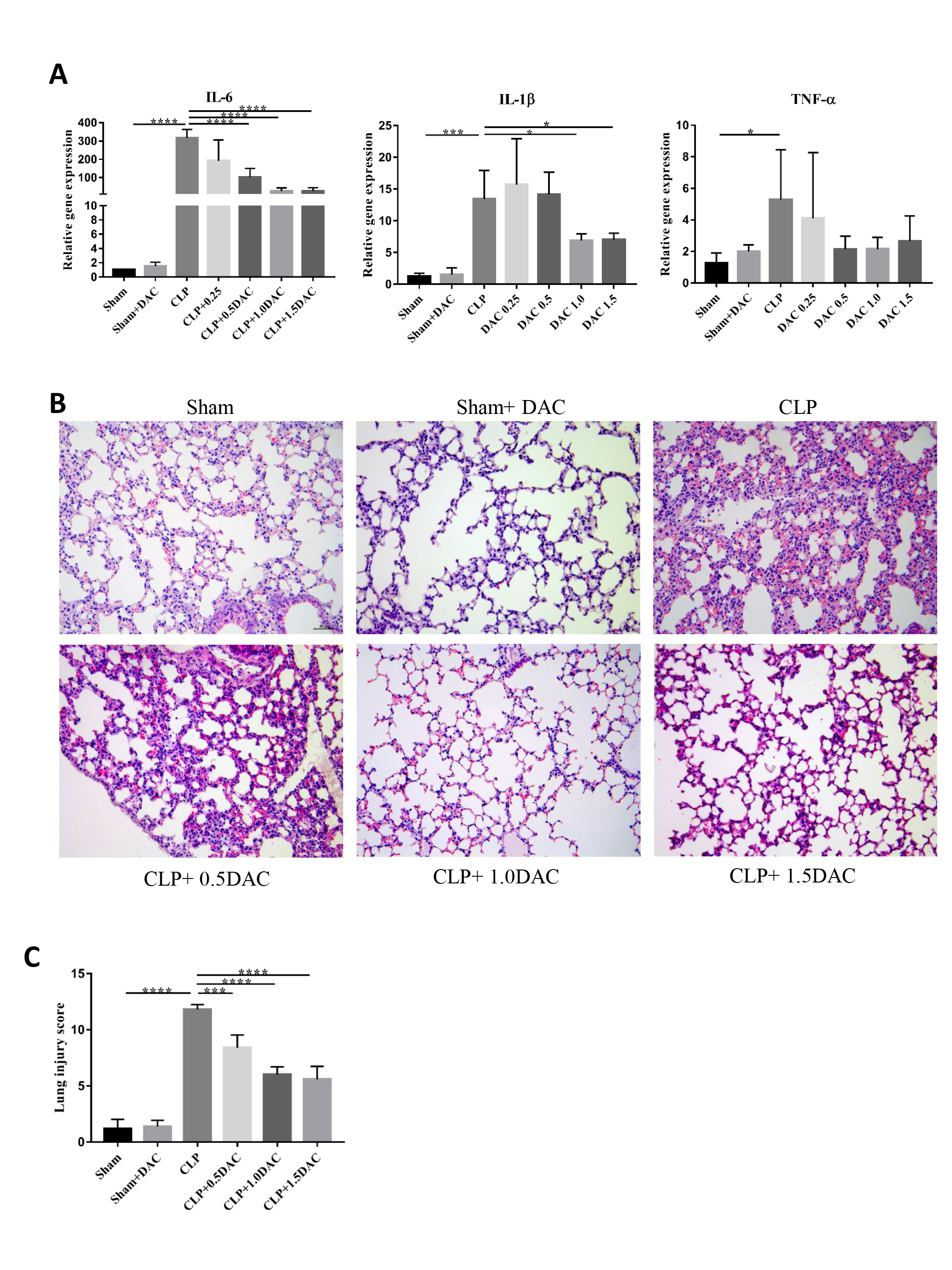

Supplement: Supplementary Figure 2 — (A) Quantitative PCR analysis of spleen IL-6, IL-1β, TNF-α in different dose of Decitabine treated mice. (B) Effects of Decitabine on CLP lung morphology. The mice were sacrificed 6 h after operation and lung tissue was stained with haematoxylin-eosin (magnification, × 400). (C) The lung injury score. n = 5. Data were shown in Mean ± SD. ****p < 0.0001, ***p < 0.001, **p < 0.01, *p < 0.05. Comparisons between two groups were done by independent student's t-test. [file Image_2.TIF]

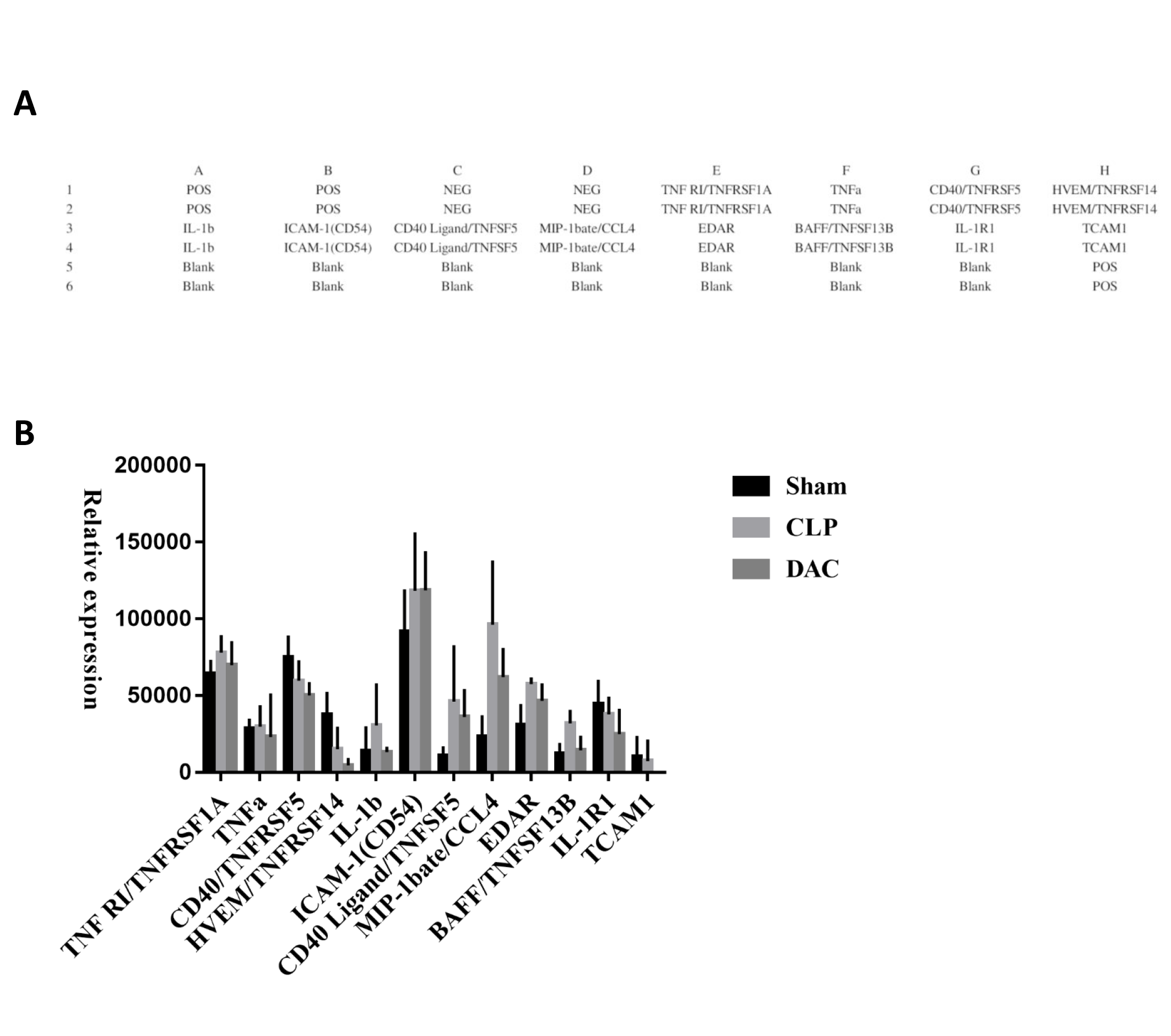

Supplement: Supplementary Figure 3 — (A) Location of cytokine antibody spotted onto the membrane. (B) Relative expression of 12 cytokines in mice spleen lysate n = 3. Data were shown in Mean ± SD. [file Image_3.TIF]

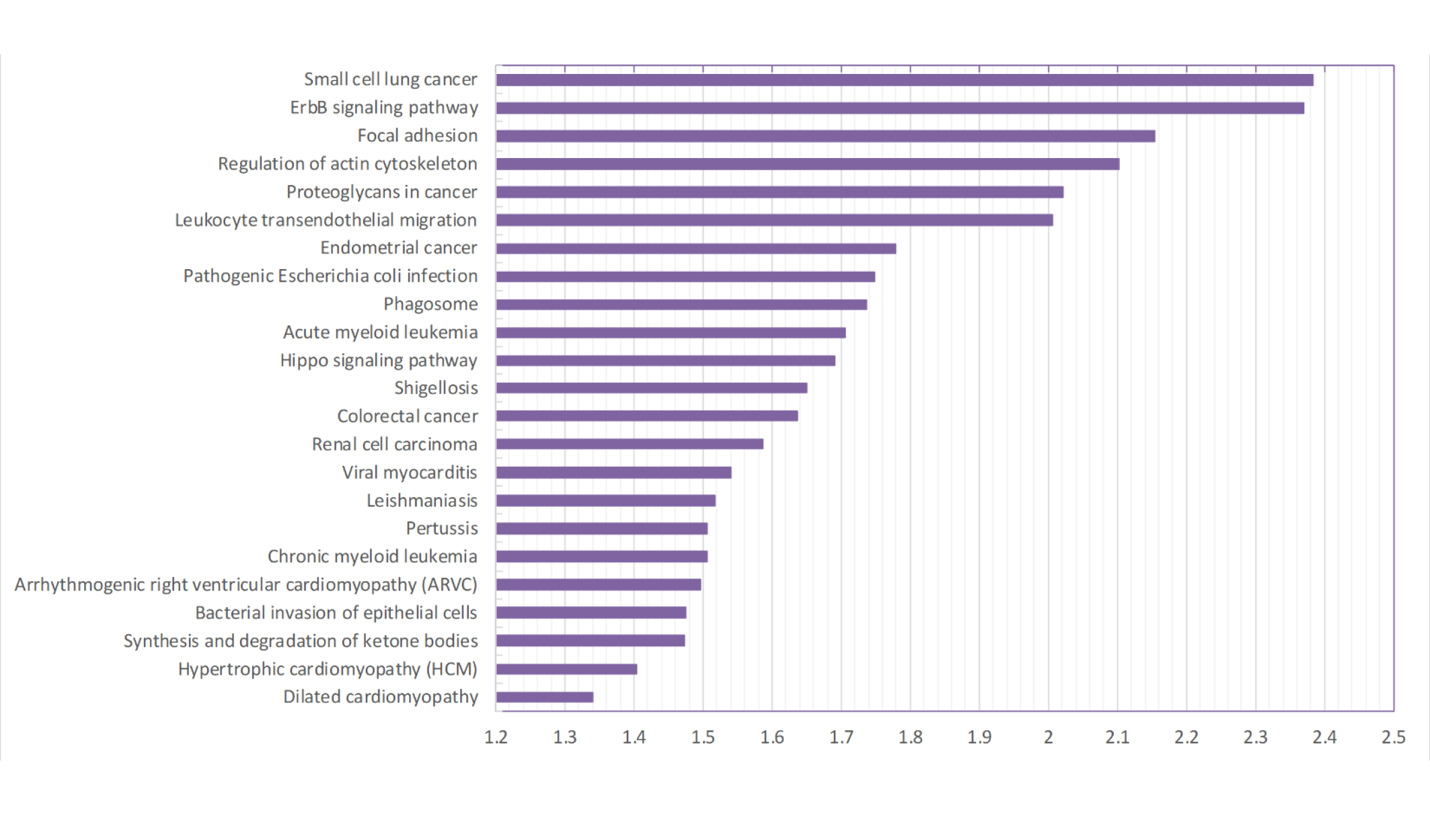

Supplement: Supplementary Figure 4 — Kyoto Encyclopedia of Genes and Genomes (KEGG) enrichment analysis of methylation variable positions (p < 0.05). [file Image_4.TIF]

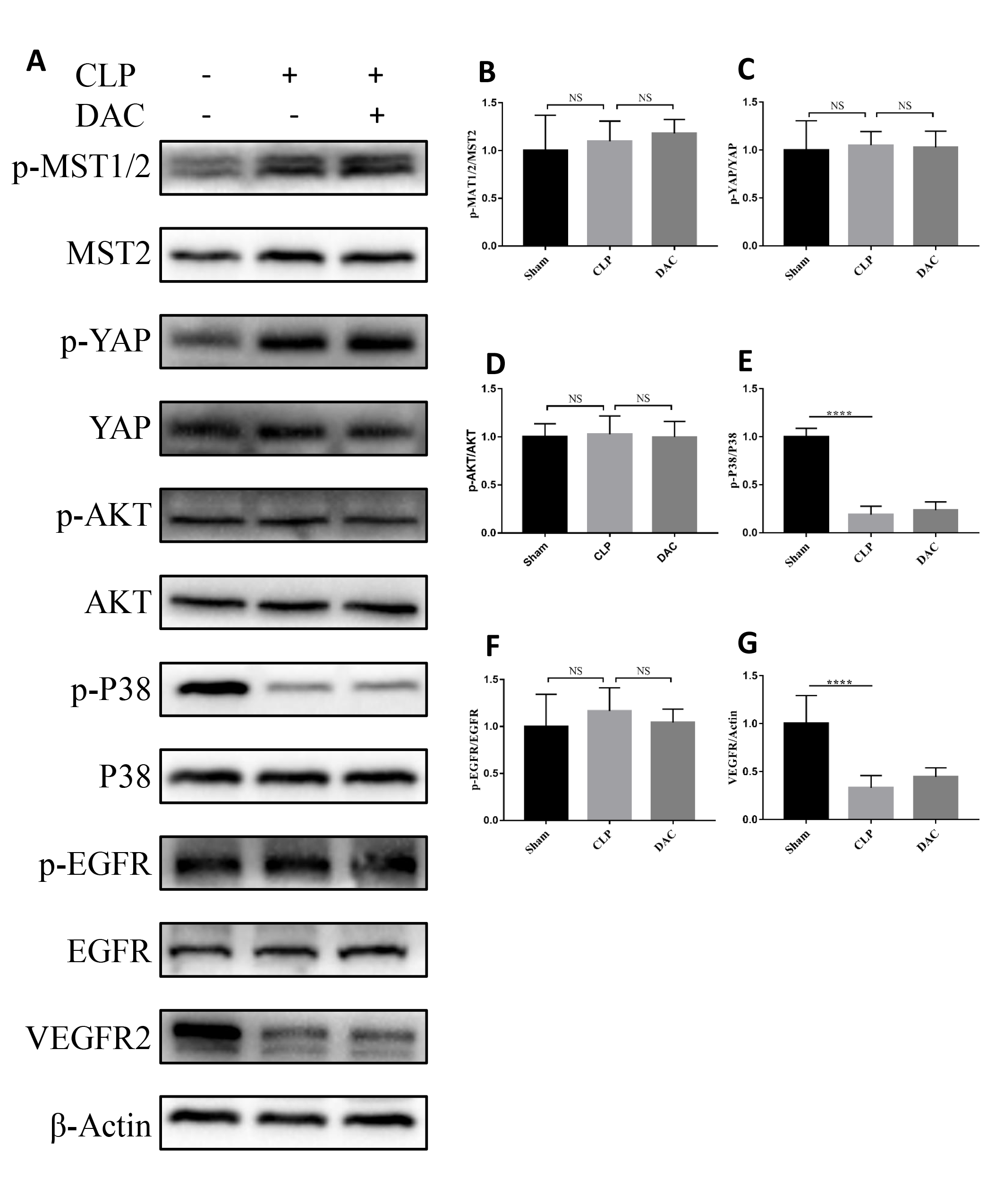

Supplement: Supplementary Figure 5 — (A) Representative western blot for p-JNK, JNK, p-MST1/2, MST2, p-AKT, AKT, p-P38, P38, p-EGFR, EGFR, VEGFR2, and β-Actin in sepsis mice spleen with or without Decitabine treatment. (B–G) The bands were quantified by densitometry and normalized to the density of corresponding total protein or β-actin n = 3.Data were shown in Mean ± SD. ****p < 0.0001. Comparisons between two groups were done by independent student's t-test. [file Image_5.TIF]
