# Supplementary material for: Inhibiting DNA Methylation Improves Survival in Severe Sepsis by Regulating NF-κB Pathway
Source: Front Immunol. 2020 Jul 2;11:1360. doi: 10.3389/fimmu.2020.01360 (PMC7343767; doi:10.3389/fimmu.2020.01360)
Supplement: Supplementary file 6 [file Table_1.docx]

**Supplement Table 1**

| mIL-1R_Q_F1 | Forward | GGGAAGCAATATCCGGTCACA |
| --- | --- | --- |
| mIL-1R_Q_R1 | Reverse | TGACGTTGCAGATCAGTTGTATC |
| mVCAM-1_Q_F1 | Forward | TTGGGAGCCTCAACGGTACT |
| mVCAM-1_Q_R1 | Reverse | GCAATCGTTTTGTATTCAGGGGA |
| mCOX2_Q_F1 | Forward | TTCAACACACTCTATCACTGGC |
| mCOX2_Q_R1 | Reverse | AGAAGCGTTTGCGGTACTCAT |
| mMIP2_Q_F1 | Forward | GCGGTCAAAAAGTTTGCCTTG |
| mMIP2_Q_R1 | Reverse | AGCCTTGCCTTTGTTCAGTATC |
| mMyD88_Q_F1 | Forward | AGGACAAACGCCGGAACTTTT |
| mMyD88_Q_R1 | Reverse | GCCGATAGTCTGTCTGTTCTAGT |
| mICAM_Q_F1 | Forward | GTGATGCTCAGGTATCCATCCA |
| mICAM_Q_R1 | Reverse | CACAGTTCTCAAAGCACAGCG |
| mMIP-1β_Q_F1 | Forward | TTCCTGCTGTTTCTCTTACACCT |
| mMIP-1β_Q_R1 | Reverse | CTGTCTGCCTCTTTTGGTCAG |
| mβ-actin_Q_F1 | Forward | GGCTGTATTCCCCTCCATCG |
| mβ-actin_Q_R1 | Reverse | CCAGTTGGTAACAATGCCATGT |
| mIL6_Q_F1 | Forward | TAGTCCTTCCTACCCCAATTTCC |
| mIL6_Q_R1 | Reverse | TTGGTCCTTAGCCACTCCTTC |
| mIL-1β_Q_F1 | Forward | AGTTGACGGACCCCAAAAG |
| mIL-1β_Q_R1 | Reverse | AGCTGGATGCTCTCATCAGG |
| mTNF-α_Q_F1 | Forward | CATGAGCACAGAAAGCATGATCCG |
| mTNF-α_Q_R1 | Reverse | AAGCAGGAATGAGAAGAGGCTGAG |
